# Plant probiotic bacteria *Bacillus* and *Paraburkholderia* improve growth, yield and content of antioxidants in strawberry fruit

**DOI:** 10.1038/s41598-018-20235-1

**Published:** 2018-02-06

**Authors:** Mosaddiqur Rahman, Abdullah As Sabir, Julakha Akter Mukta, Md. Mohibul Alam Khan, Mohammed Mohi-Ud-Din, Md. Giashuddin Miah, Mahfuzur Rahman, M. Tofazzal Islam

**Affiliations:** 1 Department of Biotechnology, Bangabandhu Sheikh Mujibur Rahman Agricultural University, Gazipur, 1706 Bangladesh; 2 Department of Crop Botany, Bangabandhu Sheikh Mujibur Rahman Agricultural University, Gazipur, 1706 Bangladesh; 3 Department of Agroforestry and Environment, Bangabandhu Sheikh Mujibur Rahman Agricultural University, Gazipur, 1706 Bangladesh; 4 Extension Service, West Virginia University, Morgantown, WV 26505 USA; 5 Present Address: Department of Biological Sciences, King Abdul Aziz University, Jeddah, 21589 Saudi Arabia

## Abstract

Strawberry is an excellent source of natural antioxidants with high capacity of scavenging free radicals. This study evaluated the effects of two plant probiotic bacteria, *Bacillus amylolequefaciens* BChi1 and *Paraburkholderia fungorum* BRRh-4 on growth, fruit yield and antioxidant contents in strawberry fruits. Root dipping of seedlings (plug plants) followed by spray applications of both probiotic bacteria in the field on foliage significantly increased fruit yield (up to 48%) over non-treated control. Enhanced fruit yield likely to be linked with higher root and shoot growth, individual and total fruit weight/plant and production of phytohormone by the probiotic bacteria applied on plants. Interestingly, the fruits from plants inoculated with the isolates BChi1 and BRRh-4 had significantly higher contents of phenolics, carotenoids, flavonoids and anthocyanins over non-treated control. Total antioxidant activities were also significantly higher (*p* < 0.05) in fruits of strawberry plants treated with both probiotic bacteria. To the best of our knowledge, this is the first report of significant improvement of both yield and quality of strawberry fruits by the application of plant probiotic bacteria BChi1 and BRRh-4 in a field condition. Further study is needed to elucidate underlying mechanism of growth and quality improvement of strawberry fruits by probiotic bacteria.

## Introduction

The consumption of fresh fruits and vegetables containing bioactive compounds has increased considerably in recent years. Several lines of evidence suggest that these bioactive compounds are beneficial to human health^1^. Strawberry (*Fragaria* × *annanasa*) is an excellent source of natural antioxidants including carotenoids, vitamins, anthocyanins, phenols, and flavonoids with the capacity of scavenging free radicals^2,3^. Major phenolic compounds such as flavonoids present in strawberries exhibited high antioxidant^4^ and anticancer properties^5^. The level of flavonoid groups, flavonols, and anthocyanins in strawberries are directly or indirectly linked with total antioxidant capacity^6^. Elevated levels of these secondary metabolites should provide better health benefits to the consumers of strawberry. Use of synthetic chemicals for enhancing fruit yield and contents of secondary metabolites in strawberry fruits is discouraged due to the health concern of the consumer and deleterious effects to the environment. Therefore, a novel eco-friendly approach is preferred to improve yield and quality of strawberry fruits.

Plant probiotic bacteria are naturally occurring plant-associated microorganisms that enhance the growth of the host plants including yield, and may suppress diseases when applied in adequate amounts^7^. Major genera of plant growth promoting probiotic bacteria include *Bacillus, Paraburkholderia, Pseudomonas, Acinetobacter, Alcaligenes, Arthrobacter*, and *Serratia*^8–12^. They provide beneficial effects to host plants through production of phytohormones, antibiotics and lytic enzymes, fixation of atmospheric nitrogen, solubilization of soil mineral nutrients and induction of systemic resistance in the host plants^7^. Although promotion of growth and yield of strawberry by plant growth promoting probiotic bacteria has been described in several reports^13–15^, no information is available on the influence of plant probiotics on contents of anthocyanins, carotenoids, flavonoids, total phenolics and antioxidant activities of strawberry fruits. Recently, enhancement of vitamin C content in strawberry fruits by the application of a strain of the genus *Phyllobacterium* has been reported^16^. We recently developed a bank of 650 endophytic plant probiotic bacteria isolated from diverse plant species of Bangladesh. Application of some of these bacteria significantly increased growth and yield of several crops under nutrient poor conditions^11^. Screening and field testing of our 650 plant probiotic bacteria assisted us identifying the most effective isolates. Among the effective ones, *Bacillus amyloliquefaciens* BChi1 and *Paraburkholderia* BRRh-4^11^ showed multiple plant growth promoting traits including solubilization of phosphorus, production of phytohormone and antagonism to major phytopathogens^11,12,15,17^. *Paraburkholderia* is a newly proposed genus delineated from *Burkholderia* through phylogenomic analysis^12^. We examined the phylogenic position of *Burkholderia* BRRh-4 based on its 16S rRNA gene sequence and renamed it as *Paraburkholderia fungorum*^12^. Considering the potential of these plant probiotics for enhancing plant growth, yield and quality, this study was conducted to investigate the performance of BChi1 and BRRh-4 on growth, fruit yield and biofunctional properties of field-grown strawberry fruits. Specific objectives of the study were to (i) evaluate the effects of two plant probiotic bacterial strains BChi1 and BRRh-4 on growth and fruit yield of strawberry, and (ii) determine the effects of these probiotic bacteria on contents of total anthocyanins, carotenoids, flavonoids, phenolics and antioxidant activities in fresh strawberry fruits.

## Results

### Enhancement of growth and yield of strawberry by plant probiotic bacteria

#### Leaf canopy characteristics

Application of probiotic bacteria significantly (*p* < 0.05) enhanced leaf length (cm), leaf width (cm), leaf number/plant and canopy diameter compared with non-treated control (Table 1). The highest leaf length (23.42 cm) was observed in plants treated with BRRh-4 and the lowest (18.68 cm) was in non-treated control plants. Similar to leaf length, leaf width also significantly varied among the treatments. The highest leaf width (14.15 cm) was found in plants treated with BRRh-4 whereas lowest leaf width (10.78 cm) was observed in non-treated plants. Leaf number and canopy diameter of strawberry plants also varied significantly among the treatments (Table 1). The highest leaf number (18.21)/plant was recorded in BChi1 treated plants and lowest leaf number/plant (13.84) was observed in non-treated control plants. Consistent with leaf number, canopy diameter also had significant variation among the treatments. The highest canopy diameter (33.75 cm) was recorded in BChi1 treated plants and lowest canopy diameter (29.12 cm) was observed in non-treated control.

**Table 1:** Effect of plant probiotic bacteria on leaf length, leaf width, leaf number/plant and canopy diameter (cm) of cv. Strawberry Festival. Mean values within a column followed by the same letter do not differ significantly by Fisher’s protected LSD test at (*p* ≤ 0.05). Data are presented as mean ± SE (n = 24).

Treatment	Leaf length (cm)	Leaf width (cm)	Leaf number/plant	Canopy diameter (cm)
Control	18.68 ± 0.71b	10.78 ± 0.81b	13.84 ± 0.40b	29.12 ± 0.48b
*Bacillus amyloliquefaciens* BChi1	23.12 ± 0.28a	13.54 ± 0.15a	18.21 ± 0.60a	33.75 ± 0.60a
*Paraburkholderia fungorum* BRRh-4	23.42 ± 0.52a	14.15 ± 0.22a	17.46 ± 1.10a	33.39 ± 1.28a

#### Plant height and root length

**Figure 1:**
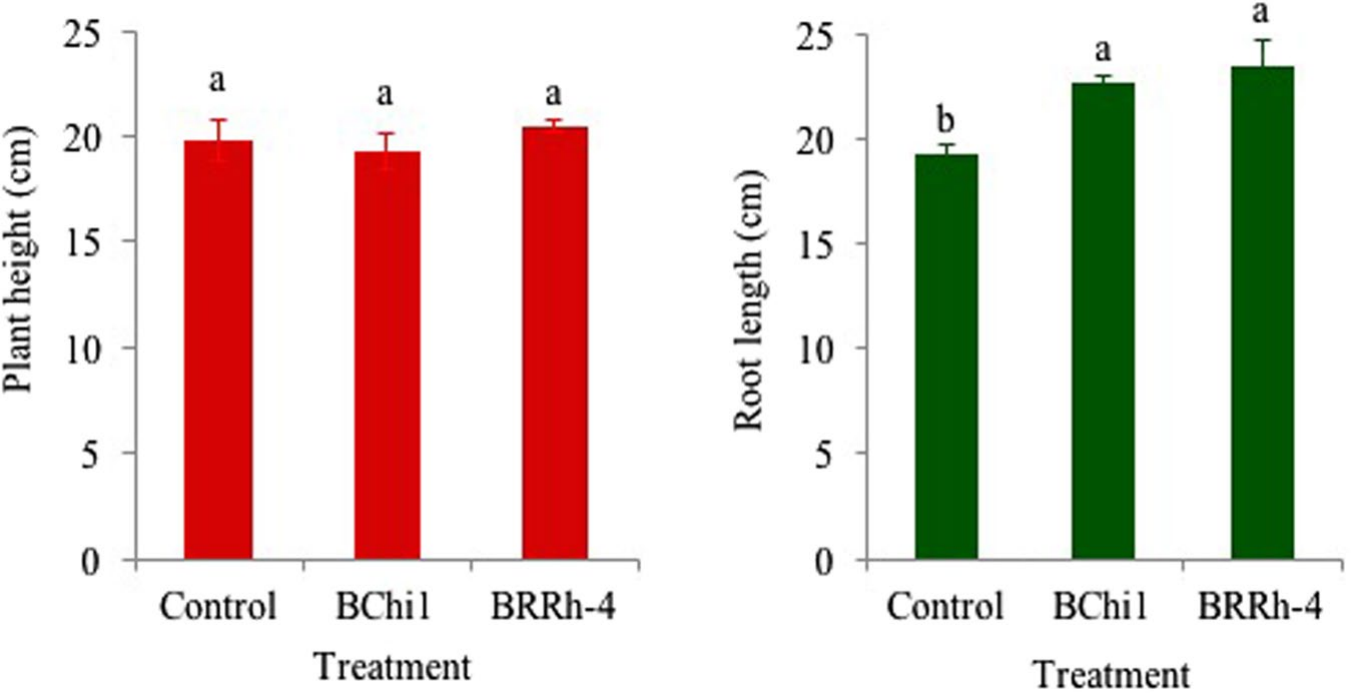
Average plant height (cm) and root length (cm) of cv. Strawberry Festival as influenced by the application of plant probiotic bacterial strains BChi1 and BRRh-4. Mean values in the bars followed by the same letter(s) are not significantly different as assessed by Fisher’s protected LSD (least significance difference) at p ≤ 0.05.

Plant height and root length also were positively influenced and varied significantly due to the plant probiotic bacteria applications. The highest plant height (20.50 cm) was observed in BRRh-4 treated plants whereas lowest was recorded in non-treated control (18.58 cm) plants (Fig. 1). Similar to plant height, root length also significantly (*p* < 0.05) varied among the treatments (Fig. 1). The highest root length (23.5 cm) was found in BRRh-4 bacteria treated plants and lowest root length (19.25 cm) was recorded in non-treated control plants (Fig. 1).

#### Effect of probiotic bacteria on fresh and dry biomass

The plant probiotic bacteria had positive effects on fresh and dry biomass of strawberry plants (Table 2). The highest shoot fresh weight was recorded in BRRh-4 (220 g/plant) treated plants, which was statistically superior to all other treatments, and lowest shoot fresh weight was found in non-treated control (134.5 g/plant) plants. However, this difference of biomass diminished due to drying up of plant samples. The highest shoot dry weight was found in BRRh-4 (49.6 g/plant) treated plants and lowest was found in non-treated control (30.7 g/plant) plants. Consistent with shoot fresh and dry weight increase, application of probiotic bacteria significantly influenced root fresh and dry weight of strawberry plants (Table 2). The highest root fresh weight was found in BRRh-4 (21 g/plant) bacteria treated plants whereas the lowest root fresh weight was recorded in non-treated control (12.63 g/plant). The highest root dry weight was recorded in BRRh-4 (11 g/plant) treated plants whereas the lowest root dry weight was found in non-treated control (7.2 g/plant) plants (Table 2). There were no significant differences between the growth enhancement of strawberry roots by two plant probiotic bacteria.

**Table 2:** Effects of plant probiotic bacteria on fresh and dry biomass of cv. Strawberry Festival. Mean values within a column followed by a common letter do not differ significantly by Fisher’s protected LSD test at (*p* ≤ 0.05). Data are means ± SE (n = 24).

Treatment	Shoot fresh weight (g/plant)	Shoot dry weight (g/plant)	Root fresh weight (g/plant)	Root dry weight (g/plant)
Control	134.5 ± 0.7c	30.7 ± 0.8b	12.6 ± 0.3b	7.2 ± 2.2b
*Bacillus amyloliquefaciens* BChi1	193.2 ± 2.8b	49.0 ± 2.3a	20.5 ± 0.8a	10.8 ± 3.4a
*Paraburkholderia fungorum* BRRh-4	220.0 ± 2.9a	49.6 ± 0.7a	21.0 ± 1.2a	11.0 ± 3.2a

#### Effect of probiotic bacteria on fruit yield

**Figure 2:**
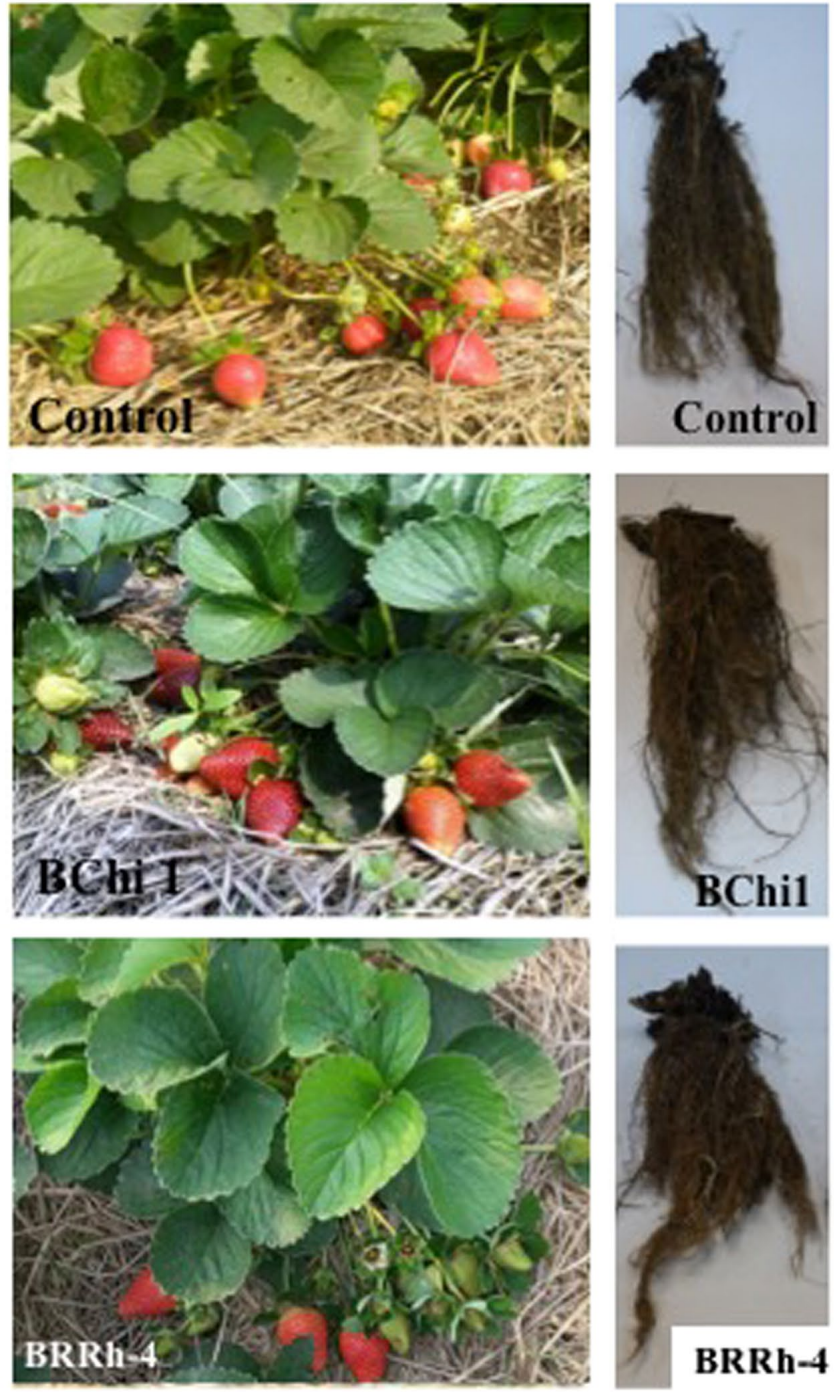
Effect of plant probiotic bacterial strains BChi1 and BRRh-4 on vegetative, reproductive and root growth of cv. Strawberry Festival. Photos of vegetative and reproductive growth were taken at the beginning of fruit harvest whereas root photos were taken at the end of the season when fruit harvest was complete.

**Figure 3:**
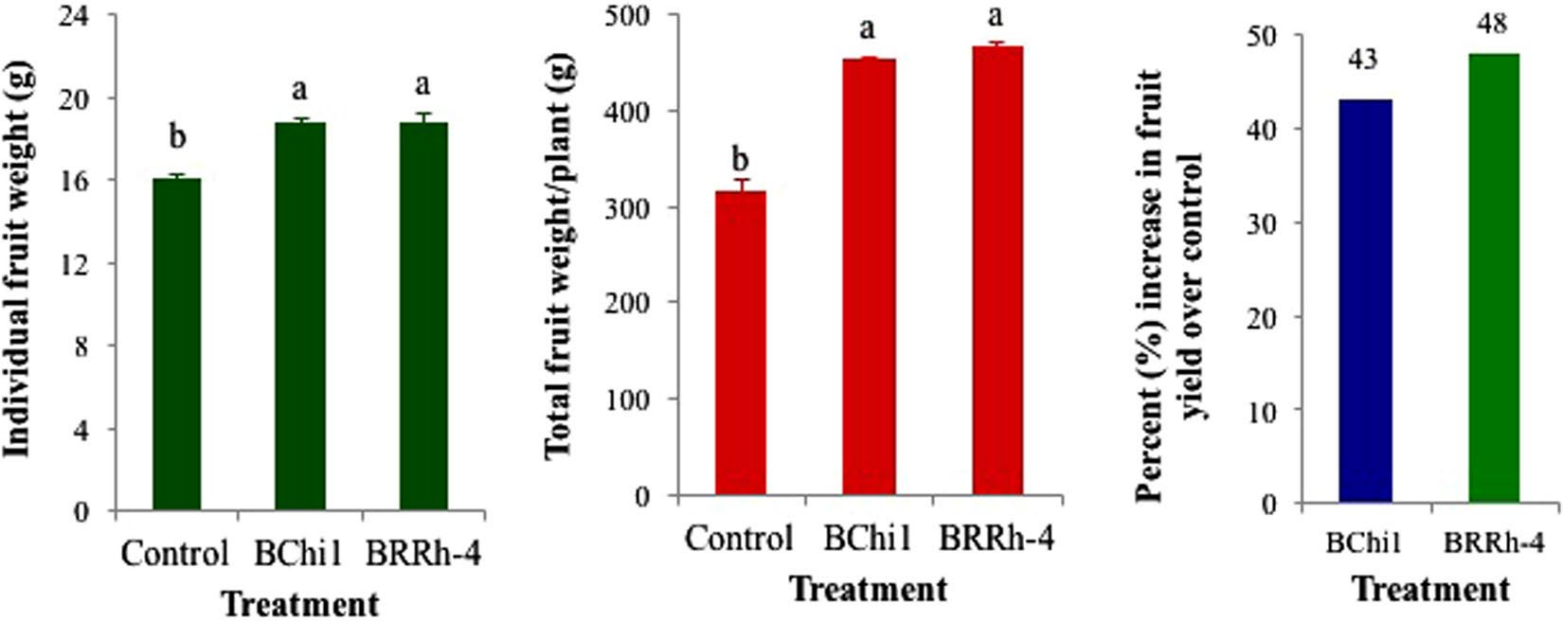
Average individual (g), total fruit weight (g/plant) and increase in fruit yield of cv. Strawberry Festival over control by the application of plant probiotic bacterial strains BChi1 and BRRh-4. One way ANOVA was performed for analysis of the data and mean values in the bars followed by the same letter(s) are not significantly different as assessed by Fisher’s protected LSD (least significance difference) at p ≤ 0.05.

Consistent with several important growth parameters of strawberry plants recorded in this study, enhancement of fruit yield of strawberry was significantly influenced by probiotic bacteria. Plant probiotic bacteria significantly increased individual fruit weight and fruit yield of strawberry compared with non-treated control. Both individual fruit weight (g/fruit) and total fruit weight per plant (g/plant) were higher in plant probiotic bacteria (BRRh-4 and BChi1) treated plants than non-treated control plants (Fig. 2). Higher individual fruit weight (~18.8 g/fruit) was recorded in both BChi1 and BRRh-4 treated plants compared with 16.1 g/fruit in non-treated control plant (Fig. 3). The highest strawberry fruit yield was obtained by the treatment of BRRh-4 (467.8 g/plant) followed by BChi1 (453.0 g/plant), and the lowest (316.6 g/plant) was in non-treated control (Fig. 3). There was no significant difference between fruit yield of strawberry obtained by the treatment of plant probiotic bacterial strains BRRh-4 and BChi1. The BRRh-4 and BChi1 treated plants had about 48% and 43% higher fruit yield compared to non-treated control plants (Fig. 3).

### Enhancement of antioxidant contents and total antioxidant activities of strawberry fruits by plant probiotic bacteria

#### Total anthocyanin and carotenoids

**Figure 4:**
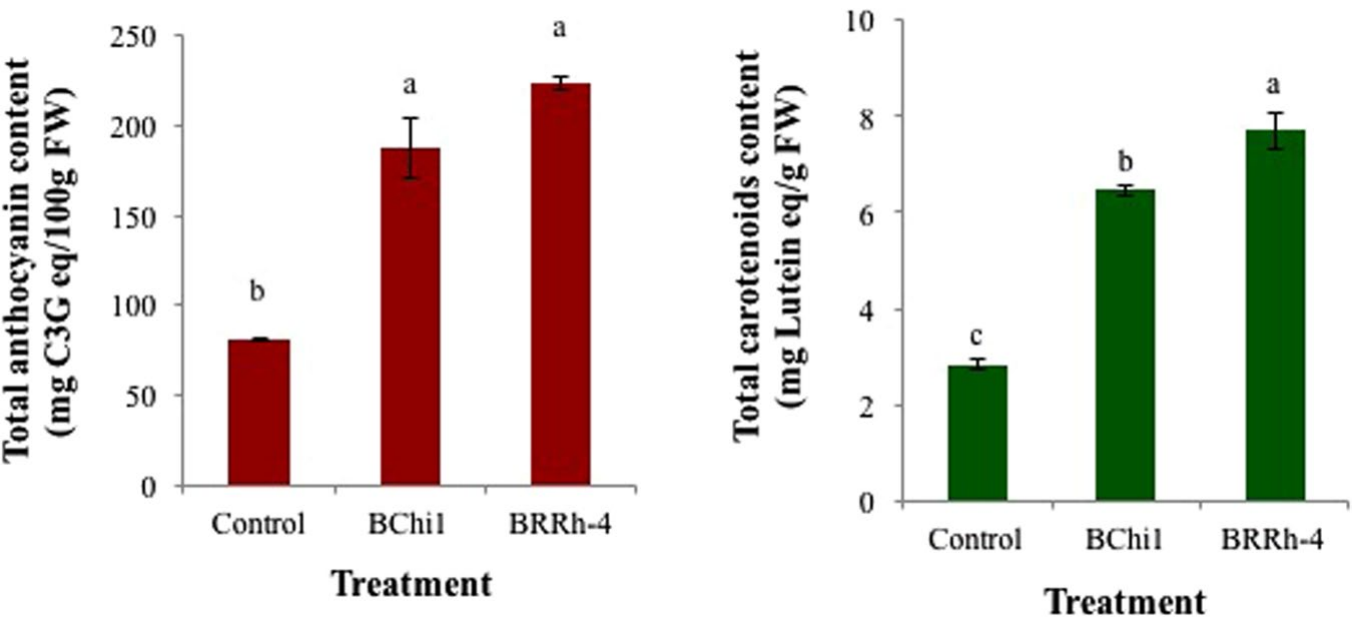
Enhancement of total anthocyanin (mg cyanidin-3-*O*-glucoside equivalent/100 g FW) and carotenoids [mg (lutein)/g FW] contents in cv. Strawberry Festival by the treatment of plant probiotic bacteria. One way ANOVA was performed for analysis of the data and mean values in the bars followed by the same letter(s) are not significantly different as assessed by Fisher’s protected LSD (least significance difference) at p ≤ 0.05.

Application of plant probiotic bacteria significantly increased total anthocyanin content in strawberry fruits compared to non-treated control. The highest anthocyanin content (222.0 mg cyanidin-3-*O*-glucoside/100 g fruit) in strawberry fruits was recorded in plants treated with BRRh-4 followed by BChi1 (187.47 mg cyanidin-3-*O*-glucoside /100 g fruit) that were statistically different from non-treated control (81.11 mg cyanidin-3-*O*-glucoside/100 g fruit) (Fig. 4). Total anthocyanin contents in strawberry fruits produced by the treatments of probiotic bacterial strains BRRh-4 and BChi1 were statistically similar. Similar to anthocyanin, total carotenoids content in strawberry fruits from plants treated with plant probiotic was also significantly higher than those of non-treated plants. The highest carotenoids content was estimated in fruits of BRRh-4 (7.71 mg lutein/g fruit) treated plants, which was statistically different from the fruits of BChi1 (6.46 mg lutein/g fruit) and non-treated control plants (2.82 mg lutein/g fruit) (Fig. 4).

#### Total flavonoids and phenolics

**Figure 5:**
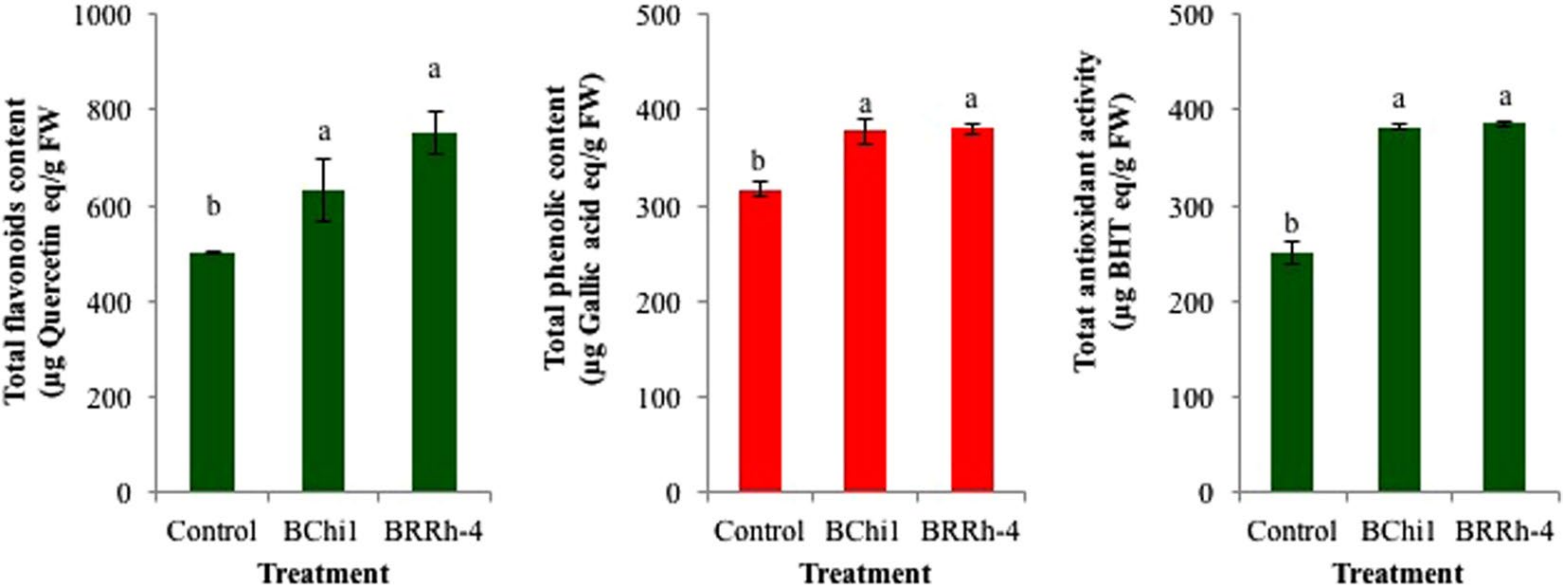
Enhancement of total flavonoids (µg quercetin equivalent/g FW), phenolics (µg gallic acid equivalent/g FW) contents and antioxidant activity (µg BHT equivalent/g FW) of fresh cv. Strawberry Festival by the treatment of plant probiotic bacteria. One way ANOVA was performed for analysis of the data and mean values in the bars followed by the same letter(s) are not significantly different as assessed by Fisher’s protected LSD (least significance difference) at p ≤ 0.05.

Total flavonoids content of strawberry fruit significantly varied with the application of plant probiotic bacteria compared to non-treated control. Plants inoculated with strain BRRh-4 had the highest total flavonoids content (751.81 µg Quercetin/g fruit) in fruits, while the lowest total flavonoids content (501.03 µg Quercetin/g fruit) was recorded in non-treated control (Fig. 5). Fruits produced in plants treated with BChi1 had 631.98 µg Quercetin/g fruit total flavonoids, which was statistically similar to the fruits produced in BRRh-4 treatment. Total phenolics content in fruits was also significantly enhanced by the application of plant probiotic bacteria compared to non-treated control. The fruits produced by the plants treated with BRRh-4 had the highest total phenolics content (380.5 µg Gallic acid/g fruit), which was statistically similar to the phenolics content in BChi1 (377.72 µg Gallic acid/g fruit) treatment (Fig. 5). The lowest total phenolics content (317.08 µg Gallic acid/g fruit) was recorded in fruits produced in non-treated control plants.

#### Total Antioxidant activity

To evaluate whether plant probiotic bacteria had any effect on antioxidant activities of strawberry fruits obtained from both probiotic bacteria and non-treated control plants, we estimated total antioxidant activities of fresh strawberry fruits by DPPH assay. The results of the DPPH assay for total antioxidant activity were expressed as butylated hydroxytoluene (BHT) equivalents per gram of strawberry fruit. As expected, the total antioxidant activity of fresh strawberry fruits was highest in BRRh-4 (385.47 µg BHT/g fruit) followed by BChi1 treatment (382.00 µg BHT/g fruit) (Fig. 5). Total antioxidant activities of the fruits produced by the treatment of both probiotic bacteria were significantly higher than that of non-treated control (250.89 µg BHT/g fruit).

## Discussion

Alternative approach for plant growth promotion and pest management is being explored for sustainable agriculture worldwide. Application of synthetic chemical inputs (fertilizer and pesticides) in crop production has created both environmental and health hazards^18^. This is more relevant and significant for fruit crops such as strawberry that are used for fresh consumption^19^. This study explored an environment-friendly option for boosting strawberry plant growth, fruit yield and functional properties of fruits through the application of two plant growth promoting probiotic bacteria and compared the results with that of non-treated control. Results showed significant improvement in plant growth, yield, various antioxidant contents and total antioxidant activities of strawberry fruits by the application of both *Bacillus amylolequifaciens* BChi1 and *Paraburkholderia fungorum* BRRh-4 treatment compared to non-treated control. Although plant growth promotion by the application of various species of *Bacillus* and *Paraburkholderia* has been reported^10,12,20^, this study for the first time demonstrated that two plant probiotic bacteria, *B. amylolequifaciens* BChi1 and *Paraburkholderia fungorum* BRRh-4 significantly increased both yield and functional properties of strawberry fruits. This study is one of the few of this kind to evaluate the effects of plant probiotics on both yield and quality of strawberry fruits. Significant increase in vitamin C contents in strawberry fruits by the application of a strain of genus *Phyllobacterium* has recently been reported^16^.

One of the interesting findings of this study is that both plant probiotic bacteria significantly improved growth and yield of strawberry almost at the same level with some minor differences although they belong to different bacterial genera. Probiotic bacterium, BRRh-4 provided the highest fruit yield increase (48%) in plants of ‘Strawberry Festival’ compared to non-treated control (Fig. 3). Generally, plant growth promoting rhizobacteria facilitate plant growth directly by either assisting in resource acquisition (nitrogen, phosphorus and essential minerals) or modulating plant hormone levels, or indirectly by inhibiting various pathogens as biocontrol agents^21^. Early colonization of root system has the potential to preclude pathogen colonization and infection in addition with induction of disease resistance or a range of beneficial secondary metabolites. However, a large-scale field study with multiple cultivars is needed for testing this hypothesis. Although suppression of fungal pathogens that cause black root rot complex of strawberries was not the focus of this study, it is possible that due to pre-colonization of strawberry root system by beneficial bacteria, treated plants had lower root disease. Future studies should evaluate roots of treated and non-treated plants for the extent of black root rot complex/crown rot. Probiotic bacteria also promote plant growth by a number of similar mechanisms regardless of their taxonomic groupings. These include phosphate solubilization activity^22,23^, indole acetic acid production^24^ and production of siderophore^25^. Moreover, a number of other beneficial effects on plant growth have been attributed to plant probiotic bacteria that include modification of root morphology, enhanced uptake of minerals and alteration of nitrogen accumulation and metabolism and/or induction of gene expression in host plants^26^.

In the current study, inoculation of strawberry plants separately with two bacterial isolates significantly increased vegetative growth (leaf length, leaf number, shoot and root dry weights) of the strawberry plants (Tables 1 and 2). Similar growth enhancement effects by bacteria on growth and yield of crop plant were reported in several earlier studies^27–29^. Due to the documented growth promoting effect by two plant probiotic bacteria on strawberry in this study, it is logical to get these included in the PGPR group. The growth-promoting activities of PGPR on plants can be explained in various ways, including biocontrol and induction of disease resistance in the inoculated plant, biological N_2_ fixation, phosphorus solubilization, and/or production of phytohormone such as IAA^24,28^. The objective and extent of work in this study did not allow us to determine if phytohormones or nutrient uptake were also enhanced by probiotic bacteria, but we have undertaken a separate study to assess those beneficial effects, which is highly likely to occur. We recently demonstrated a significant increase in growth and yield of rice by *Paraburkholderia fungorum* BRRh-4^11^ and *B. amylolequifaciens* BChi1 under nutrient deficient soils^11,17^. Application of these probiotic bacteria through root colonization and spray application on strawberry plants enhanced growth, yield and functional properties of fruits and the improvement was modest that may benefit strawberry growers and consumers if adopted in their production practice.

Strawberry is particularly a good source of antioxidants with 10-fold greater capacity of scavenging free radicals than that of many other fruits, including oranges, kiwis, grapefruits, grapes and mangoes^30^. Anthocyanins are in part responsible for the antioxidant property of strawberry fruits, together with ascorbic acid and a wide variety of phenolics, including hydroxybenzoic and hydroxycinnamic acid derivates, flavanols, proanthocyanidins and hydrolysable tannins^31^. In the current study, application of plant probiotic bacteria significantly increased total antioxidants, carotenoids, flavonoids, phenolics and total anthocyanin contents in fresh strawberry fruits compared to non-treated control (Figs 4 and 5), making these very interesting and important findings. Treatments of strawberry plants with bacteria strains BRRh-4 and BChi1 consistently produced higher antioxidants, carotenoids, flavonoids, phenolics and total anthocyanins compared to non-treated control. Previous study showed that the members of the genus *Phyllobacterium* were good plant probiotics with the capacity of increasing fruit yield as well as quality^32^. However, this study for the first time demonstrated that both *B. amyloliquefaciens* and *Paraburkholderia fungorum* not only increased yield but also significantly improved contents of several antioxidants and total antioxidant activities of fruits. Functional properties of fruits are dependent on bioactive compounds that may be increased when plants are biofertilized with *Rhizobium*^33^ by impacting plant’s secondary metabolism. Chamam *et al*. showed that *Azospirillum* sp. was able to modulate the phenolic compounds in rice^34^. The effect of arbuscular mycorrhizal colonization on the concentration of anthocyanins was previously measured in strawberry fruits by Castellanos-Morales *et al*.^35^. They showed for the first time that symbiosis induced an increase in cyanidin-3-*O*-glucoside (and that of some other phenolics). Both *B. amyloliquefaciens* and *Paraburkholderia fungorum* are well-known and widely studied plant growth promoting bacteria, and many of the strains are used for producing commercial products^10,12,36,37^. Molecular mechanisms of plant growth promotion by *Bacillus* spp. have largely been elucidated^10,36^. Growth promotion of common bean by *Paraburkholderia fungorum* has also been reported^12,20^. To the best of our knowledge, there are no data available on the positive effects of plant probiotic bacteria *B. amyloliquefaciens* and *Paraburkholderia fungorum* on enhancement of total antioxidants, carotenoids, flavonoids, phenolics and total anthocyanins in strawberry. In the current study, vitamin C content in strawberry fruits also increased by both of these probiotic bacteria (data not shown). In an earlier study, increased induction of transcriptional profile of phenylpropanoid pathway genes and increased contents of flavonoid and lignin in *Arabidopsis* leaves in response to the application of commercial microbial products have been reported^38^.

Results from this study suggest that application of *B. amyloliquefaciens* and *Paraburkholderia fungorum* not only can significantly increase growth and fruit yield but also enhance functional properties of strawberry by inducing enhanced production of total antioxidants, carotenoids, flavonoids, phenolics and anthocyanins. Elucidation of molecular mechanism involved with the improvement of growth, yield and quality of strawberry by these two plant probiotic bacteria would help finding more efficient microbial strains for inducing gene expression in plants related to secondary metabolites overproduction. The findings of this study however, indicate the plant probiotic bacteria BRRh-4 and BChi1 isolated from the native environment could be used as natural agents for sustainable production of high quality strawberry with no or little additional use of expensive synthetic inputs.

## Methods

### Experimental site and treatment application

This study was conducted at the experimental field of Bangabandhu Sheikh Mujibur Rahman Agricultural University during the period of November 30, 2014 to March 25, 2015. Soil of the experimental field was shallow red brown terrace under Salna Series^39^ in Madhupur Tract (Agroecological zone 28) having a pH 6.71. This soil contained 1.70% organic matter, 0.115% nitrogen (N), 21.35 ppm phosphorus (P) and 0.24 meq. 100 g^−1^ soil exchangeable potassium (K). Runner grown seedlings of cv. Strawberry Festival were collected from the Akafuji Agrotechnology, Rajshahi, Bangladesh. All intercultural operations and fertility management were performed following commercial recommendations.

### Experimental design and layout

The field experiment was laid out in a randomized complete block design (RCBD) with three replications. The unit plot size was 75 cm × 150 cm and the plants were spaced 30.5 cm × 30.5 cm on the beds. Beds were raised 30.5 cm above main field with 34 inches aisles in between 2 beds. Each plot contained 8 plants in two adjacent rows 30.5 cm apart. Thirty days old strawberry plug plants were transplanted on November 30, 2014. Data on vegetative growth and fruit yield were collected from each plant of each replicated plot.

### Bacterial isolates used in the experiment

**Figure 6:**
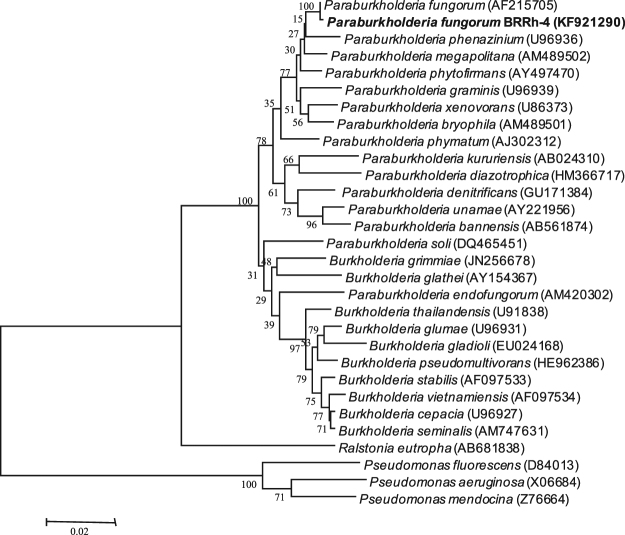
A neighbor-joining phylogenetic tree based on the 16S rRNA gene sequences showing the position of the isolate *Paraburkholderia* sp. BRRh-4 (NCBI GenBank accession No. KF921290) to other strains of the genus *Burkholderia*. Accession numbers for the 16S rRNA sequences used for each organism are provided in the brackets following the name of the organism. The tree was rooted using one species from the genus *Ralstonia* and three species from the genus *Pseudomonas*. The significance of each branch is indicated by a bootstrap value based on 1000 replications.

The bacterial strains BChi1 and BRRh-4 used in this study were isolated as endophytes from the surface sterilized roots of chili plant and rhizosphere soil of rice cv. BINAdhan-7, respectively. They were tentatively identified through 16S rRNA gene sequencing as *Bacillus amyloliquefaciens* BChi1 (GenBank accession No. KT306960) and *Burkholderia* sp. BRRh-4 (GenBank accession No. KF921290)^11^. However, a recent comparative molecular signature and phylogenomic analyses of a large number of *Burkholderia* spp. from diverse origins suggest that non-pathogenic environmental *Burkholderia* should be considered as a new genus *Paraburkholderia*^12^. Reexamination of 16S rRNA sequence data of BRRh-4 revealed 99% similarity to the sequence data of the *Paraburkholderia fungorum* (AF215705). Therefore, we renamed BRRh-4 as *Paraburkholderia fungorum* BRRh-4 (NCBI Genbank accession No. KF921290). We constructed a neighbor-joining (NJ) phylogenetic tree based on 16S rRNA sequences (Fig. 6). All the sequences utilized in the study were obtained from GenBank of NCBI. Multiple sequence alignment was carried out using the CLUSTAL W Multiple Alignment program in BioEdit version 7.2.3, and a NJ tree based upon 1000 bootstrap replicates of this alignment was constructed using the Kimura 2-parameter model in MEGA 6.0.

Both of these plants-associated bacteria (BChi1 and BRRh-4) were confirmed to possess antagonistic activities towards major phytopathogenic microorganisms, produced indole-3-acetic acid and displayed high phosphate solubilizing activities. They were preserved in 20% glycerol at −20 °C until used in this study.

### Preparation and application of bacterial suspension

Bacterial isolates (BRRh-4 and BChi1) were cultured separately in 500 mL nutrient broth (Merck, Germany) in conical flasks taking a single colony from actively growing bacterial culture plates that were maintained on lab bench at ambient temperature (25° ± 2 °C). Then each flask was placed on a shaking incubator adjusted at 120 rpm and 25 °C for 72 h for bacterial growth in nutrient broth. The broth was centrifuged at 12,000 g and the pellet was washed thrice with sterilized distilled water to remove nutrients. The bacterial pellet was suspended in water and diluted to a concentration of approx. 1 × 10^9^ CFU mL^−1^. Roots of strawberry plug plants were dipped overnight in the suspension to facilitate bacterial root colonization^13^. For foliar application, nutrient-free bacterial suspension containing ca. 1 × 10^9^ CFU mL^−1^ of bacteria was sprayed until run-off in five different times with 10-d intervals starting (at flower initiation stage) from December 20, 2014 to February 22, 2015. Plants in the non-inoculated control plots were sprayed with equal volume of sterile water without any bacterial cells.

### Assessment of probiotic bacterial effect on vegetative growth of strawberry plants

A total of three treatments, plant inoculation with strain BChi1, BRRh-4 and non-inoculated control (8 plants/treatment) were assessed from three replicated plots. Effect of probiotic bacteria on strawberry vegetative growth was determined by measuring plant height (cm), number of leaves per plant, leaf length (cm) leaf width (cm), and canopy diameter (cm) with a ruler. The appearance of new flowers and fruits was recorded weekly. Runners generated from the growing plants were removed immediately as noticed on any plant. Fruits were harvested when most of the fruit surfaces had turned red. Sepals were discarded and only fruit flesh was used for biochemical analyses^40^. The length and diameter of the fruits were recorded; then fruits were weighed and immediately stored at −20 °C for analyses. After 20 weeks of growth, plants were dug out of the ground, root systems and shoots were separated, washed under running water, and weighed. Length of roots (cm) was recorded with a ruler. Shoots and roots were dried at 60 °C for one week to assess dry weight.

### Determination of total anthocyanins, carotenoids, flavonoids, and phenolics

Sample matrix, particle size and binding of phenolics with other biochemical substances such as carbohydrate and proteins strongly influence phenolic extraction from plant materials^41^. In our assays, we chose the protocols that have been widely used due to their reproducibility. For the determination of total anthocyanins, carotenoids, flavonoids, phenolics and total antioxidant activities, 100 g preserved fruits were thawed and homogenized in a standard food homogenizer. Sub-samples were used to determine bioactive contents for each of the above listed compounds.

Anthocyanin was extracted by placing 1g of homogenized fruit sample in 5 mL 6M HCl: H_2_O: MeOH (7: 23: 70) in the dark at 4 °C for 24 h. To separate chlorophylls from anthocyanins, 2 mL of chloroform and 1 mL of water was added to 2 mL of fruit extracts and then centrifuged for 15 min at 5,000 g. Three mL of the supernatant was used for determination of total anthocyanins^42^. Anthocyanins concentration was determined as cyanidin-3-*O*-glucoside equivalent using the absorbance A530 (CT60, UV-visible spectrophotometer, PG INSTRUMENTS) and a molar extinction coefficient of 30000 l mol^−1^cm^−1^ ^43^. To determine carotenoid content, 5 mL acetone was added to 2 g homogenized fruit sample in a glass vial followed by incubation for 24 h in dark at 4 °C. Three mL supernatant was taken in a glass cuvette and the absorbance of the acetone extract was measured at 444 nm using acetone as blank in the spectrophotometer mentioned above in triplicate. Total carotenoid content was measured in mg g^−1^ of sample as lutein equivalent according to the protocol described earlier^44^.

Total flavonoid was determined spectrophotometrically according to aluminium chloride colorimetric assay method. Briefly, 0.4 mL 5% sodium nitrate was added to 1 mL methanol extract of strawberry fruit in a test tube. For blank reaction, 1 mL methanol was taken instead of methanol extract of strawberry. After 5 minutes, 0.6 mL of 10% AlCl_3_.6H_2_O was added to the mixture. At 6^th^ minute, 2 mL of 1M NaOH was added to the mixture, followed by shaking thoroughly and measuring the absorbance of the solution at 510 nm against the blank sample^45^. The measurements were compared to a standard curve of quercetin solutions and total flavonoids content was expressed as (µg/g FW) quercetin equivalent. Total phenolic compounds were also determined spectrophotometrically following Folin-Ciocalteau method. Briefly, 0.5 mL 10% (0.2 N) Folin–Ciocalteau reagent was added to each test tube containing 1 mL methanol extract of fruit sample and 1 mL methanol alone as blank. The test tubes were shaken for 10 s, covered and incubated for 15 min at room temperature. Aqueous 700 mM sodium carbonate (Na_2_CO_3_) solution (2.5 mL) was added to each reaction mixture and then vortexed, covered and incubated at room temperature for 2 h. The absorbance of the solution was measured at 765 nm against the blank sample^46^. The measurements were compared to a standard curve of gallic acid solutions and total phenolics were expressed as (µg/g FW) gallic acid equivalent.

### Antioxidant activity (DPPH radical scavenging assay)

Measurement of radical scavenging activity using discoloration of 1,1-diphenyl-2- picrylhydrazyl radicals (DPPH radical scavenging assay) has been widely used due to its stability, simplicity, and reproducibility. Therefore, antioxidant activity of strawberry fruits was assayed by DPPH (CalBiochem, Germany) radical scavenging assay^47^. The DPPH assay method is based on the reduction of DPPH, a stable free radical. The free radical of DPPH has an odd electron, which gives a maximum absorption at 517 nm (purple color). Methanol extracted supernatant of strawberry fruit sample (1mL) was taken in test tube and DPPH solution (0.0788g of 0.2 mM DPPH in 1L methanol) was added to it. The reaction mixture was incubated at 25 °C for 5 min, after which time the absorbance was measured at 517 nm^47^. When the antioxidants react with DPPH, the DPPH is reduced to DPPH-H and, as a consequence, the absorbance decreases. DPPH-H formation results in decolorization (yellow color) with respect to the number of electrons captured. The DPPH solution with corresponding solvents (i.e., without plant material) served as the control. Methanol with the respective plant extracts was used as the blank. The DPPH radical scavenging activity of each plant extract was calculated as the percentage inhibition.

1 $$ \% \text{Inhibition}\,{\rm{of}}\,{\rm{DPPH}}\,{\rm{radical}}\,{\rm{activity}}=\frac{A\,Control-A\,Sample}{A\,Control}\times 100$$

### Data collection and statistical analyses

In order to determine the effect of probiotic bacteria on plant growth, fruit yield and quality, leaf length (cm), leaf width (cm), plant height (cm), canopy diameter (cm), leaf number/plant, shoot fresh and dry weight (g), root fresh and dry weight (g), root length (cm), fruit weight (g)/plant individual fruit weight (g) and anthocyanin mg (cyanidin-3-*O*-glucoside)/100g FW, carotenoids mg (lutein)/g FW, flavonoids µg (quercetin) /g FW, phenolics µg (gallic acid)/g FW, antioxidant µg (BHT) /g FW were recorded from all plants/composite fruit samples from an experimental unit. Individual and total fruit yield of strawberry were calculated as g/fruit and g/plant, respectively. The SPSS version 16 was used for analysis of variance of the data collected from three replicates of each treatment of the experiment. Treatment means were separated using Fisher’s protected LSD test at (*p* ≤ 0.05).

### Ethical Statement

The lab and field experiment in this study were carried out following guidelines and recommendations of “Biosafety Guidelines of Bangladesh” published by Ministry of Environment and Forest, Government of the People’s Republic of Bangladesh (2005). However, the research works were strictly supervised by an advisory committee of research works of M.R. with the approval of Dean, Graduate studies, BSMRAU. The advisory committee monitored the research works considering the ethical issues.

### Availability of Data

Data used in this manuscript will be available to the public.
